# 30004 Examining Opioid Technical Assistance (TA) Requests for Hard-to-Reach Populations

**DOI:** 10.1017/cts.2021.617

**Published:** 2021-03-31

**Authors:** Holly Hagle, Michael Knabel, Michele Baker, Laurie Krom, Aimee Campbell, Frances Levin, Kathryn Cates-Wessel

**Affiliations:** 1 University of Missouri - Kansas City (UMKC); 2 Columbia University; 3 American Academy of Addiction Psychiatry (AAAP)

## Abstract

ABSTRACT IMPACT: Analyzing the types of technical assistance (basic, targeted or intensive) provided by the Opioid Response Network (ORN) to unique and hard-to-reach populations (UHRP) informs addiction health services and translational research by identifying technical assistance needs in these populations which may require a higher level of intensity. OBJECTIVES/GOALS: To improve ORN dissemination and implementation efforts, the project classifies TA requests into one of three categories: basic, targeted, and intensive. This TA Framework assists the ORN project team in understanding the level of TA required in the delivery of evidence-based practices to address opioids with communities with respect to UHRP. METHODS/STUDY POPULATION: TA requests from April 1, 2019, to April 1, 2020, were selected. The ORN classifies TA requests in one of three categories: basic (dissemination & brief consultation), targeted (services to enhance readiness and capacity), and intensive (full incorporation of innovation considering context, culture, and linguistics) (Fixsen, et. al., 2009; Becker, et al., 2020). Unique and hard-to-reach populations (UHRP) are defined based on physical location (i.e., remote or isolated), social position, or other vulnerabilities (i.e. member of an ethnic or racial minority group) (Thurman, & Harrison, 2020). ORN classifies 26 types of UHRP these types are not mutually exclusive. A frequency analysis of the UHRP types was conducted. Bivariate correlations between UHRP types that had a minimum of 30 cases were performed. RESULTS/ANTICIPATED RESULTS: Among 746 TA requests selected, 226 had missing information about UHRP types and 29 had missing information TA levels. These requests were excluded from the frequency analysis. The three most common UHRP types were people living in rural or remote areas (n=262, 50%), people who are uninsured or underinsured (n=162, 31%), and people who inject drugs (n=158, 30%). Most TA requests were targeted (69%), 23% were intensive, and 9% were basic. Bivariate correlations were performed between 21 UHRP types. Moderate (Pearson’s r=0.4-0.6) or strong correlations (r>0.6) were found for 11 occurrences for the UHRP type of ‘LGBT’, 8 for ‘Mental Illness’, and 7 for ‘Veterans’. Strong correlations were found between ‘Justice Involved’ and ‘Incarcerated’ (r=0.645), and between ‘Disabilities’ and ‘Chronic Pain’ (r=0.603). DISCUSSION/SIGNIFICANCE OF FINDINGS: There were more TA requests at targeted and intensive levels than basic levels suggesting the need for services to enhance readiness and build capacity. The moderate/strong correlations indicate that UHRP types were likely to coexist with other types. Future research can explore combining UHRP types that have moderate/strong correlations.

